# Growth-Rate Dependence Reveals Design Principles of Plasmid Copy Number Control

**DOI:** 10.1371/journal.pone.0020403

**Published:** 2011-05-27

**Authors:** Stefan Klumpp

**Affiliations:** 1 Max Planck Institute of Colloids and Interfaces, Potsdam, Germany; 2 Center for Theoretical Biological Physics, University of California San Diego, La Jolla, California, United States of America; Michigan State University, United States of America

## Abstract

Genetic circuits in bacteria are intimately coupled to the cellular growth rate as many parameters of gene expression are growth-rate dependent. Growth-rate dependence can be particularly pronounced for genes on plasmids; therefore the native regulatory systems of a plasmid such as its replication control system are characterized by growth-rate dependent parameters and regulator concentrations. This natural growth-rate dependent variation of regulator concentrations can be used for a quantitative analysis of the design of such regulatory systems. Here we analyze the growth-rate dependence of parameters of the copy number control system of ColE1-type plasmids in *E. coli.* This analysis allows us to infer the form of the control function and suggests that the Rom protein increases the sensitivity of control.

## Introduction

One aim of systems biology is the quantitative characterization of simple genetic circuits and regulatory elements in order to understand or design more complex circuitry built with these elements [1], [2], [3]. A complication in this research program is that genetic circuits are never completely isolated from the physiological state of the host cell, which, for example, provides the machinery for gene expression. The coupling of gene expression and the physiological state of the cell has recently been studied quantitatively for bacterial systems [4], [5], where the most important characteristics of the physiological state of a cell is the growth rate [6] and where growth-rate dependencies are known for many cellular parameters [7], [8]. As a result of the growth-rate dependence of cellular parameters such as cell size, gene copy number, availability of transcription and translation machinery etc., the level of expression of a gene also becomes growth-rate dependent. Changes in gene expression then reflect combinations of specific up- and down-regulation and global effects due to changes of the growth rate [4]. In addition, growth-rate dependent gene expression can mediate feedback if the growth rate is dependent on the expression level of a gene and generate bistability with subpopulations growing with different growth rates [4], [9].

Growth-rate effects are particularly pronounced for genes on plasmids, as the plasmid copy numbers can exhibit strong growth-rate dependencies [4]. This effect was used in a recent study to generate oscillations in the cell density [10]. These growth-rate dependent effects apply not only to engineered circuits hosted on the plasmid, but also to the plasmid's native circuits such as its replication control system, on which we focus in this study. Plasmid replication control is usually based on negative control by a plasmid-encoded regulatory protein or RNA, such that an excess of plasmid copies results in suppression of further plasmid replication [11], [12].

Here we use the growth-rate dependent modulation of plasmid copy number control to extract quantitative information about the replication control circuit from the growth-rate dependence of cellular and plasmid parameters. We focus on one of the best-studied examples, the ColE1-type replication control system of plasmid pBR322 in *E. coli*, which is based on the suppression of replication by a regulatory RNA called RNA I. We first show that the growth-rate dependent data for parameters of plasmid replication control as obtained in experiments of several labs over the last 25 years is consistent with a simple mathematical model for copy number control that represents the essential core of previous more detailed modeling studies [13], [14], [15], [16] and that, in the form used here, has been studied systematically by Paulsson and Ehrenberg with respect to stochastic effects and plasmid stability [17]. We derive a complete set of the parameters for this model at different growth rates (Table 1).

**Table 1:** Growth-rate dependence of parameters related to plasmid copy number control.

Parameter	Symbol	Growth rate μ [doublings/hour]					Notes and references
		0.6	1.0	1.5	2.0	2.5	
Doubling time [min]	τ	100	60	40	30	24	
Mass per cell [OD_460_ units/10^9^ cells]	*M_C_*	0.85	1.49	2.5	3.7	5.0	[8]
Cell volume [µm^3^]	*V*	0.27	0.48	0.8	1.2	1.6	a
Plasmid copy number (per cell)	*P*	39	41	46	51	55	[22]
Plasmid concentration[µM]	*p*	0.24	0.14	0.095	0.071	0.057	*p = P/V*
RNA I/Plasmid	*s/p*	0.72	1	1.22	1.30	1.20	relative to μ = 1 dbl/hr [22]
	*s/p*	6.4	9.0	10.9	11.6	10.7	Absolute values calculated using values for α_I_ and β at 1 dbl/hr
RNA I concentration [µM]	*s*	1.52	1.29	1.05	0.81	0.62	Calculated from *s/p* and *p*
Transcription rate of RNA I [min^-1^]	α_I_	7.27	11.35	14.5	15.8	15.2	[30] b
Transcription rate of RNA II [min^-1^]	α_II_	2.23	2.73	2.84	2.17	1.49	[30] b
Degradation rate of RNA I [min^-1^]	β	1.26	1.26	1.26	1.26	1.26	c
Plasmid copy number for rom^-^ relative to WT	*p* (*rom^ ¯^*)/*p*(WT)	3.2	2.2	1.75	1.5	1.4	[21], see Methods

a. Estimated from *M*_C_ using *V* = *c*×*M*_C_, based on the observation that cell mass and volume have the same growth-rate dependence (discussed in ref. [4]), using *c* = 0.2 µm^3^/(OD_460_ units/10^9^ cells) [34]

b. Lower values of the transcription rates have been reported for a different strain (2 min^-1^ for RNA I and 0.33 min^-1^ for RNA II at 0.9 dbl/hr) [28].

c. The degradation rate has been reported to be independent of growth rate [25]. The absolute value used here is measured at 0.9 dbl/hr [22]. There are considerable differences between RNA I degradation rates measured in different labs, smaller values have been reported, ∼0.35 min^-1^, independent of growth rate [25] and ∼0.3–1.4 min^-1^, growth-rate dependent [26], see Text and Figure 3B.

Based on these parameters, we then address two central aspects of a quantitative description of the control system that remain unknown despite the long history of quantitative analysis of this system: the form of the control function and the role of the Rom protein. The control function characterizes the relation between the plasmid replication rate and the RNA I concentration and determines the sensitivity of control. The form of this function is the key difference between different control models that have been proposed, in particular, the hyperbolic [13] and the exponential model [16], which are based on different assumptions about the microscopic kinetics of control [17]. Despite the central role of the control function, its form remains unknown as it is difficult to determine in direct experiments [18]. In models of the Paulsson-Ehrenberg type, the sensitivity of control has therefore been an unknown free parameter [17]. Here we determine the form of the control function from the growth-rate dependent parameter data and thereby obtain an estimate of the sensitivity of control. This estimate supports the exponential model over the hyperbolic model.

The Rom (or Rop) protein is a second plasmid-encoded control element. Rom is known to enhance the effect of the main regulator, RNA I [19], but it has also been hypothesized that Rom might also increase the sensitivity of control [11], [20]. That hypothesis has also not been tested experimentally, because such a test would again require measuring the control function. We use growth-rate dependent data for a Rom deletion strain [21] to obtain an estimate for the control function in the absence of Rom. This analysis indicates that Rom indeed increases the sensitivity of control.

## Results and Discussion

**Figure 1:**
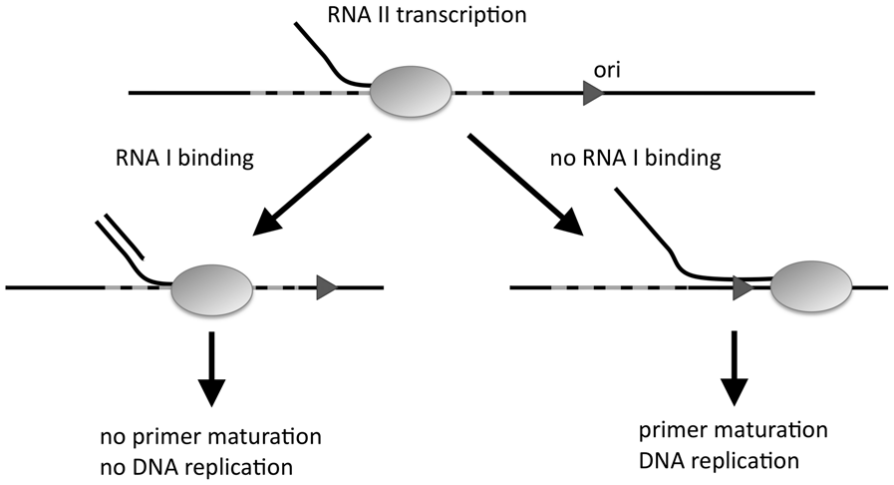
Replication control of ColE1-type plasmids. The first step of plasmid replication is the transcription of a replication primer, RNA II. If the replication regulator, RNA I, binds to the nascent RNA II within the ‘inhibition window’ (dashed area) upstream of the replication origin (ori), maturation of the primer is inhibited and DNA replication is suppressed. Otherwise, the primer matures and DNA replication can initiate.

Plasmid replication of ColE1-type plasmids is initiated by the transcription of the replication primer, RNA II. Whether transcription of RNA II results in plasmid replication depends on the concentration of the replication regulator RNA I, which inhibits the maturation of the replication primer (Figure 1). RNA I is encoded on the plasmid and has a short life time, so its concentration provides an almost instantaneous measure of the plasmid concentration [11].

We describe the replication control system with a simple and well-characterized mathematical model [16], [17]. This model consists of two equations for the dynamics of the plasmid concentration (*p*) and the concentration of RNA I (*s*) that account for transcription and degradation of RNA I, transcription of the primer, RNA II, the decision about replication, and plasmid dilution due to cell growth, see Methods. The decision about plasmid replication is characterized by the control function *R(s)*, a relation between the probability that primer transcription results in replication and the concentration of RNA I. This central quantity will be discussed in detail below.

**Figure 2:**
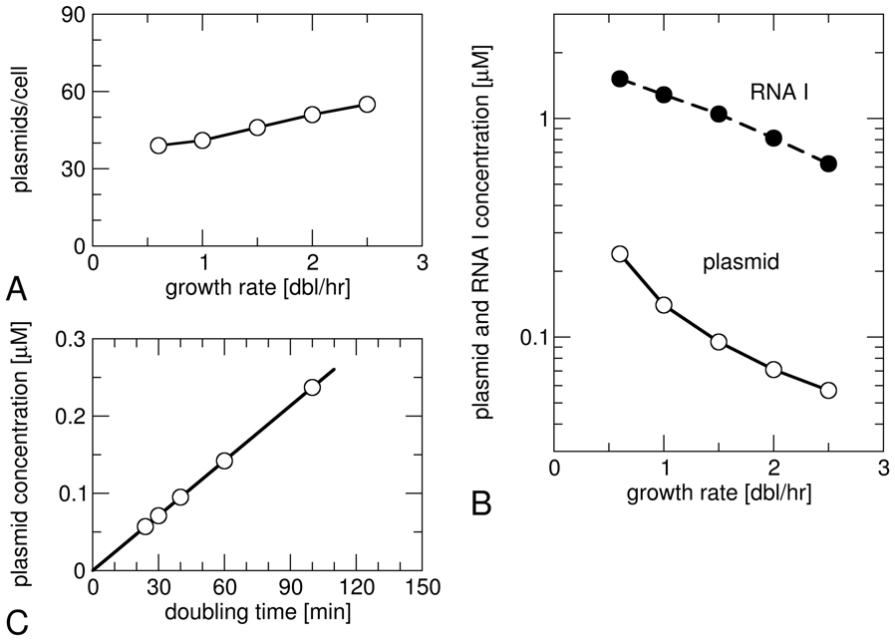
Growth-rate dependence of plasmid copy number. (A) Number of plasmid copies per cell and (B) plasmid concentration (open circles) and RNA I concentration (filled circles) as functions of the bacterial growth rate (Table 1). (C) Plasmid concentration [same data as in (B)] plotted as function of the doubling time.

Most parameters of this model as well as the steady state concentrations of the plasmid and of RNA I have been measured for the plasmid pBR322 under different growth conditions. This data has been collected in Table 1. The number of plasmid copies per cell has been found to be almost independent of growth rate (a slight increase at faster growth), but the plasmid concentration decreases strongly at fast growth (Fig. 2A and B). This decrease can be described by an approximately linear relation between the plasmid concentration and the doubling time τ (for τ<100 min, see Figure 2C) and has been observed in different strains of *E. coli* (B/r and K12) [21], [22]. It is however worth noting that rather different growth-rate dependencies have been reported for the copy numbers of plasmids that are nominally closely related (discussed in ref. [23]). Figure 2B also shows the growth-rate dependence of the concentration of RNA I, which also decreases at fast growth, but less so than the plasmid concentration its transcription rate is increased (Table 1), reflecting an increased availability of RNA polymerases [24].

**Figure 3:**
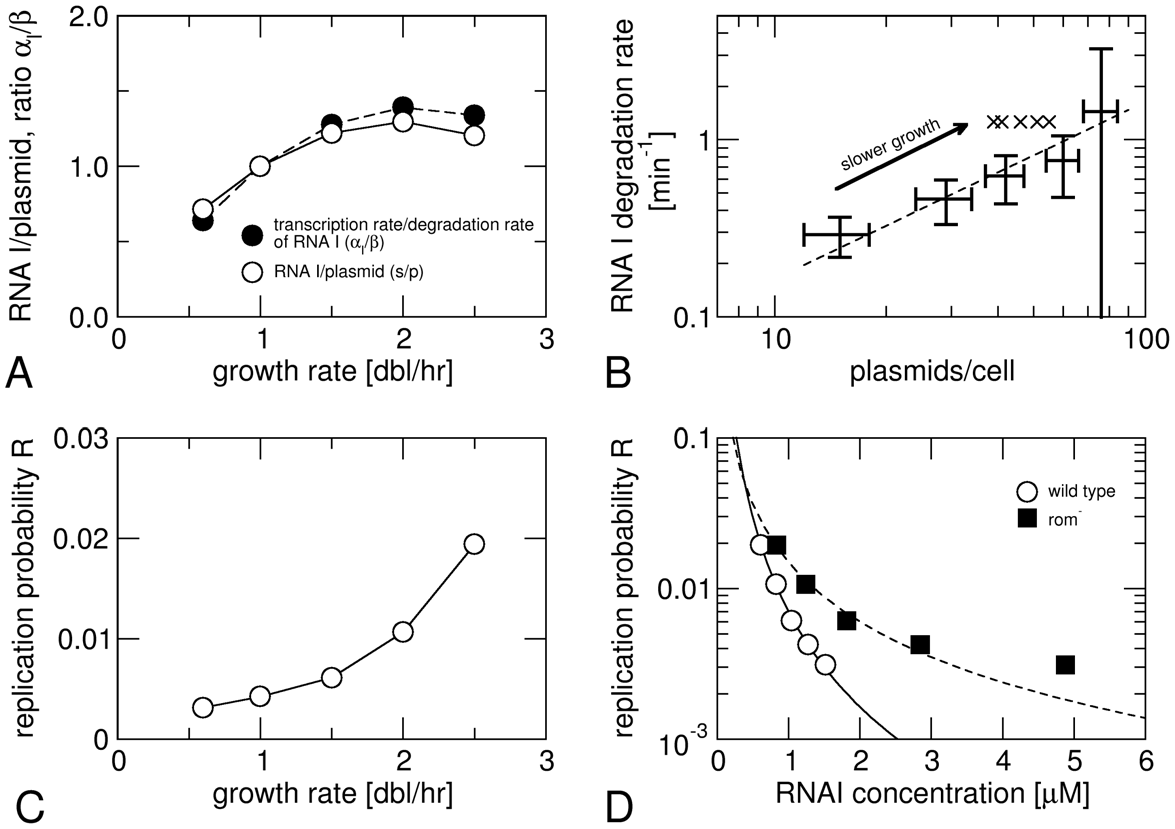
Analysis of plasmid copy number control using growth-rate dependent parameters. (A) Growth-rate dependence of the number of RNA I molecules per plasmid (open circles) and the ratio of the transcription and degradation rates of RNA I (filled circles), normalized to their values at 1 doubling/hour (data from Table 1). (B) Correlation between the plasmid copy number and the RNA I degradation rate at different growth rates for data from ref. [26], values calculated from (A) are indicated by crosses. (C) Growth-rate dependence of the replication probability *R* as calculated from Eq. (1) using data from Table 1. (D) Replication probability [as in (C)] at different growth rates plotted against the corresponding RNA I concentration for wild type and *rom* ^-^ plasmids. The lines are fits with the multiple-steps mechanism.

The model predicts that the number of RNA I molecules per plasmid (*s/p*) in the steady state is equal to the ratio of the transcription rate and degradation rate of RNA I (α_I_/β); see also Eq. 3 in Methods. This prediction is independent of the choice of the control function *R(s)*. Together with the short lifetime of RNA I [22], this relation implies that RNA I provides an almost instantaneous measure of the plasmid copy number, which is the basis of its role in suppressing copy number fluctuations [16], [17]. Using the data from Table 1, we tested this relation by plotting both quantities together in Figure 3A. The two quantities agree quite well with one another, providing support for the model. A crucial ingredient for this agreement is however that the lifetime of RNA I is independent of the growth rate as reported [25]. If we attribute the small difference between the two curves in Figure 3A to a growth-rate dependence of the degradation rate, this amounts to an effect of <30%.

Contrary to that result and to the data in Figure 2A [21], [22], a recent study reported a strong increase of the RNA I lifetime at fast growth (attributed to increased 3′ polyadenylation) and a concurrent decrease of the plasmid copy number per cell [26]. Figure 3B shows that there is a perfect correlation between the plasmid copy number and RNA I degradation rate from that study, suggesting that the decreased plasmid copy number at fast growth is due to the growth-rate dependence of RNA I degradation, consistent with the model, and that the differences between the two reports reflect real differences between the bacterial strains or the plasmids. It is likely that the difference between the strains is due to a different rate-limiting step in the pathway of degradation of RNA I, as an RNA I lacking an RNase E cleavage site [25] exhibits very similar behavior to that reported in ref. [26].

One key aspect of the present model that remains unknown is the functional form of the relation between the RNA I concentration *s* and the replication probability *R*, the probability that primer transcription results in plasmid replication. Two main scenarios have been proposed, exponential and hyperbolic replication control [17]. These two models arise from different assumptions on the microscopic kinetics of replication control: Hyperbolic control corresponds to the case where the decision about replication inhibition is governed by a single step in the pathway of primer formation [13], while the exponential scenario is obtained if that decision happens during a finite time window, which is usually associated with a ‘window of opportunity’ during the transcription of RNA II [16]. The control function *R(s)* characterizes the sensitivity of the system, which is very high for exponential control, but limited in the hyperbolic case. We calculated the replication probability *R* at different growth rates from the measured plasmid copy number and the transcription rate α_II_ of the replication primer (Figure 3C). In Figure 3D we plot the same data against the RNA I concentration for the same growth rate to obtain an estimate of the functional dependence of *R* on the RNA I concentration *s*. The latter step assumes that other factors that might influence *R* are unchanged over this range of growth rates. This assumption is believed to be correct for the Rom protein, which is available in excess [16]. It may not be valid under conditions of very slow growth or starvation, where additional regulation mechanisms such as by uncharged tRNA come into play [27]. The limited range of concentration values does not allow us to determine a unique functional form for *R(s)*, but the data appear consistent with an exponential dependence. *R(s)* clearly decreases faster than 1/*s,* which would be expected within the hyperbolic model, and thus has higher sensitivity. We fitted the data with a general control function that has an additional parameter *n* characterizing its sensitivity and that interpolates between the hyperbolic (*n* = 1) and exponential (*n*→∞) scenarios [17]. This fit results in *n*≈2.5 (solid line in Figure 3D). Figures 3C and D also show that the absolute replication probabilities are very low (∼1%) for the cellular concentration range of RNA I. Even if we use the lower values for the transcription rate of RNA II reported by Lin-Chao and Bremer [28] (Table 1), *R* is estimated to be <10%. For such low values of *R*, the control function is close to its maximal sensitivity for a given value of *n*.

To address the role of the Rom protein, a second regulator of replication, we also calculated the control function from growth-rate dependent data for plasmids that lack the *rom* gene [21], also shown in Figure 3D. Rom is usually considered as a ‘helper’ that is present in the cell in saturating amounts [16] and increases the rate of binding of RNA I to RNA II, while having no effect on their transcription and degradation rates [29]. One would therefore expect that a higher concentration of RNA I is required to repress replication of the rom^-^ plasmid compared to the wild type. Indeed, we find *s_0_n* = 47 nM for rom^-^ and 16 nM for the wild-type (dashed and solid lines in Figure 3D). In addition, Figure 3D clearly shows that the *s*-dependence of the replication probability is weaker for the rom^-^ plasmid than for the wild type, as the *R(s)* curve for rom^-^ has a smaller slope. Its functional form is not exponential and may be consistent with a hyperbolic form (the fit shown as a dashed line yields *n*≈1.4). Independent of the precise functional form of *R*(s), the comparison of the curves indicates that the Rom protein increases the sensitivity of the regulation system with respect to the RNA I concentration by making the regulation function steeper. This increase in sensitivity due to Rom is very similar to a scenario proposed by Ehrenberg [20], in which RNA I and Rom act as two independent measures of plasmid concentration for the replication decision, resulting in linear sensitivity (*n* = 1) in the absence of Rom and quadratic sensitivity (*n* = 2) in its presence.

To provide an effective control of plasmid replication, the increase in sensitivity due to Rom (to which our analysis points at the population average level) must apply to individual cell. In particular, the concentration of Rom has to provide a measure of the instantaneous plasmid concentration. Indeed the Rom concentration has been found to be proportional to plasmid concentration in a population average under different growth conditions [21], but whether this is true instantaneously and in individual cell is not known. The latter would require the Rom protein to be unstable, as assumed in some models [13], but this has not been established experimentally so far (see the discussion in ref. [18]). The proportionality of Rom and plasmid concentration at different growth rates would then also require that the lifetime of the Rom protein is independent of growth rate.

In summary, our analysis shows that using a complete set of parameters for plasmid copy number control for different steady-state growth conditions can provide information about the control mechanism such as its sensitivity with respect to the RNA I concentration and the role of the Rom protein. Obviously, the strength of the conclusions that can be drawn from such analysis is directly limited by the number of different conditions for which complete parameter sets have been determined. Therefore our analysis may be improved if data for additional growth conditions become available. From a more general point of view, this analysis provides an example of how the growth-rate dependence of the parameters of a regulatory system can be used to study the design of the control system. One advantage of this approach is that it makes use of the range of regulator concentrations (RNA I in our case) that occur naturally under different physiological conditions and does not require experimental perturbations of the control system in order to vary the regulator concentrations. This feature makes the approach particularly suited for the study of core systems of cellular regulation that are easily disrupted by experimental modifications.

## Methods

### Model for replication control

We use a commonly used model for a ColE1-type plasmid replication control system [15], [16], [17] which consists of two equations for the dynamics of the plasmid concentration *p* and of the concentration *s* of the replication regulator, RNA I,



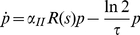
(1)




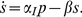
(2)Equation (2) describes the dynamics of RNA I, which is transcribed from each copy of the plasmid with rate α_I_ and degraded with rate β. Equation (1) describes the balance of plasmid replication and dilution due to cell growth with doubling time τ. The rate of plasmid replication is modeled as the transcription rate α_II_ of the replication primer, RNA II, times the probability *R*(*s*) that primer transcription results in replication. For the fit of the functional dependence of the replication probability *R*(*s*) on the concentration *s* of RNA I in Figure 3D, we use the general expression *R(s) = 1/[1+s/(n s_0_)]^n^*, which applies to a pathway where the decision about whether replication occurs can happen at multiple subsequent steps [17]. This expression contains the two main scenarios discussed in the literature, hyperbolic and exponential replication control, as limiting cases: Hyperbolic control is obtained for *n* = 1 and leads to *R(s) = 1/(1+s/s_0_)*; the exponential scenario with *R(s) = *exp*(-s/s_0_)* is obtained in the limit n→∞. The parameter *n* characterizes the maximal sensitivity of the control function, defined by the slope of the control function on a double-logarithmic scale. Maximal sensitivity is obtained for large s, where only a small fraction of replication primers leads to replication. The parameter *s_0_* defines the typical concentration scale for inhibition of replication. Microscopically it is given by the ratio of the rate constant for binding of RNA I to RNA II and the forward rate in the pathway to primer maturation [17].

The steady-state solution of these equations is given by
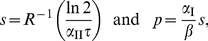
(3)where *R* ^-1^ is the inverse function of the replication control function *R(s)*. A noteworthy feature of this solution is that the concentration of the regulator RNA I does not depend on the parameter values of its own transcription and degradation rate. Rather it is fixed by the growth rate through the constraint that each copy of the plasmid needs to be replicated once per division cycle to allow for the existence of a steady-state plasmid concentration.

### Growth-rate dependent data

Data for the growth-rate dependence of the model parameters was collected from the literature and is summarized in Table 1. Where necessary, this data was interpolated to the same set of growth rates. Notes in Table 1 indicate if different values have been reported for different strains of *E. coli* or in experiments from different laboratories. In principle, it would be desirable to have all this data obtained from the same strain. Unfortunately, such data is not available; but we think that the combination of data from different sources used here is a good approximation to that ideal case. First we note that growth-rate dependencies of cellular parameters are generally rather robust [4], [5], [7], [8] with dependencies on growth rate rather than on detailed experimental conditions, although one cannot necessarily expect this to be true for expression from specific promoters. It is however very likely for the promoters of plasmid replication control, because transcription of RNA I is constitutive [30] and therefore reflects the availability of RNA polymerases [24], [30], while transcription of RNA II depends on the regulatory nucleotide ppGpp and is thus directly dependent on growth rate [30]. We therefore expect that these two transcription rates reflect global properties of the cells, which depend on the growth rate and are rather robust with respect to details of the experimental conditions. This said, we tried to use data from one source as much as possible and therefore based our study mainly on the data of the Bremer lab, which has characterized the growth-rate dependence of many parameters of *E. coli* [8], [22]. Most of that data was obtained for *E. coli* B/r strains, with the exception of the growth-rate dependence of the transcription rates of RNA I and RNA II, which were determined for a K12 strain. By the argument given above, we do not expect qualitatively different growth-rate dependence for these promoters in the two strains. Also growth-rate dependent transcription rates have been determined in both strains for some promoters (ribosomal and constitutive) and were found to be very similar [31], [32]. The data for the Rom deletion strain was taken from Ref. [21] and is also for a K12 strain. Again, we expect these results to be fully comparable to the data for the B/r strain as the parent (rom^+^) strain in Ref. [21] exhibits very similar growth rate dependence of the plasmid copy number and the ration of RNA II and RNA I as the B/r strain of Ref. [22]. Furthermore, it is known that Rom has no effect on RNA II transcription [19], [33] and on the stability of RNA I or RNA II [29], so we expect these values to be same in both strains. Ref. [21] does not provide absolute plasmid copy numbers, but the data for rom- and WT plasmids exhibit a linear relation between the ratio of rom^-^ and wild-type plasmids (taken at the same growth) and the doubling time, *p* (*rom^ ¯^*)/*p*(WT)≈τ/*A*+*B* with *A*≈41 min and *B*≈0.77. This linear relation is valid for doubling times up to approximately 100 min, and was used to estimate plasmid copy numbers for the rom^-^ strain.

### Calculation of the control function for wildtype and *rom* ^-^ plasmids

The steady-state value of the replication probability *R(s)* at a given growth rate is calculated from α_II_ and the doubling time τ according to Equation 1. The *s*-dependence of *R* is reconstructed by plotting these values against the concentrations of RNA I at the same growth rates (see Results and discussion). As the transcription rate α_II_ of RNA II, the only plasmid parameter in Equation 1, is not expected to be affected by the deletion of *rom*, the replication probability is the same for a *rom* ^-^ strain as for the wild-type plasmid at the same growth rate (Figure 3C). However the concentration *s* of the replication regulator, RNA I, and thus the functional form of *R*(*s*) are different. We estimate *s* using Eq. 2 as *s*(*rom* ^-^) = *s*(WT)*p*(*rom* ^-^)/*p*(WT), assuming that transcription and degradation rates of RNA I are unaffected by the *rom* deletion [29]. This estimate of *s* together with the replication probability in Figure 3C leads to the control function for rom^-^ plasmids in Figure 3D.

## References

[1] Andrianantoandro E, Basu S, Karig DK, Weiss R (2006). Synthetic biology: new engineering rules for an emerging discipline.. Mol Syst Biol.

[2] Guido NJ, Wang X, Adalsteinsson D, McMillen D, Hasty J (2006). A bottom-up approach to gene regulation.. Nature.

[3] Bintu L, Buchler NE, Garcia HG, Gerland U, Hwa T (2005). Transcriptional regulation by the numbers: models.. Curr Opin Genet Dev.

[4] Klumpp S, Zhang Z, Hwa T (2009). Growth rate-dependent global effects on gene expression in bacteria.. Cell.

[5] Scott M, Gunderson CW, Mateescu EM, Zhang Z, Hwa T (2010). Interdependence of cell growth and gene expression: origins and consequences.. Science.

[6] Neidhardt FC, Ingraham JL, Schaechter M (1990). Physiology of the bacterial cell: a molecular approach..

[7] Schaechter M, Maaløe O, Kjeldgaard NO (1958). Dependency on medium and temperature of cell size and chemical composition during balanced growth of Salmonella typhimurium.. Journal of General Microbiology.

[8] Bremer H, Dennis PP, Neidhardt FC (1996). Modulation of chemical composition and other parameters of the cell by growth rate.. Escherichia coli and Salmonella.

[9] Tan C, Marguet P, You L (2009). Emergent bistability by a growth-modulating positive feedback circuit.. Nat Chem Biol.

[10] Marguet P, Tanouchi Y, Spitz E, Smith C, You L (2010). Oscillations by minimal bacterial suicide circuits reveal hidden facets of host-circuit physiology.. PLoS One.

[11] Summers DK (1996). The biology of plasmids..

[12] Helinski DR, Toukdarian AE, Novick AP, Neidhardt FC (1996). Replication control and other stable maintenance mechanisms of plasmids.. Escherichia coli and Salmonella.

[13] Brendel V, Perelson AS (1993). Quantitative model of ColE1 plasmid copy number control.. Journal of Molecular Biology.

[14] Ataai MM, Shuler ML (1986). Mathematical model for the control of ColE1 type plasmid replication.. Plasmid.

[15] Keasling JD, Palsson BO (1989). On the kinetics of plasmid replication.. J Theor Biol.

[16] Brenner M, Tomizawa J (1991). Quantitation of ColE1-encoded replication elements.. Proc Natl Acad Sci U S A.

[17] Paulsson J, Ehrenberg M (2001). Noise in a minimal regulatory network: plasmid copy number control.. Q Rev Biophys.

[18] Paulsson J, Nordstrom K, Ehrenberg M (1998). Requirements for rapid plasmid ColE1 copy number adjustments: a mathematical model of inhibition modes and RNA turnover rates.. Plasmid.

[19] Tomizawa J, Som T (1984). Control of ColE1 plasmid replication: enhancement of binding of RNA I to the primer transcript by the Rom protein.. Cell.

[20] Ehrenberg M (1996). Hypothesis: hypersensitive plasmid copy number control for ColE1.. Biophys J.

[21] Atlung T, Christensen BB, Hansen FG (1999). Role of the rom protein in copy number control of plasmid pBR322 at different growth rates in Escherichia coli K-12.. Plasmid.

[22] Lin-Chao S, Bremer H (1986). Effect of the bacterial growth rate on replication control of plasmid pBR322 in Escherichia coli.. Mol Gen Genet.

[23] Kim BG, Shuler ML (1990). Analysis of pBR322 replication kinetics and its dependency on growth rate.. Biotechnol Bioeng.

[24] Klumpp S, Hwa T (2008). Growth-rate-dependent partitioning of RNA polymerases in bacteria.. Proc Natl Acad Sci U S A.

[25] Lin-Chao S, Cohen SN (1991). The rate of processing and degradation of antisense RNAI regulates the replication of ColE1-type plasmids in vivo.. Cell.

[26] Jasiecki J, Wegrzyn G (2003). Growth-rate dependent RNA polyadenylation in Escherichia coli.. EMBO Rep.

[27] Wrobel B, Wegrzyn G (1998). Replication regulation of ColE1-like plasmids in amino acid-starved Escherichia coli.. Plasmid.

[28] Lin-Chao S, Bremer H (1987). Activities of the RNAI and RNAII promoters of plasmid pBR322.. J Bacteriol.

[29] Tomizawa J (1990). Control of ColE1 plasmid replication. Interaction of Rom protein with an unstable complex formed by RNA I and RNA II.. J Mol Biol.

[30] Liang ST, Bipatnath M, Xu YC, Chen SL, Dennis P (1999). Activities of constitutive promoters in Escherichia coli.. Journal of Molecular Biology.

[31] Zhang XY, Bremer H (1996). Effects of Fis on ribosome synthesis and activity and on rRNA promoter activities in Escherichia coli.. Journal of Molecular Biology.

[32] Liang ST, Dennis PP, Bremer H (1998). Expression of lacZ from the promoter of the Escherichia coli spc operon cloned into vectors carrying the W205 trp-lac fusion.. J Bacteriol.

[33] Som T, Tomizawa J (1983). Regulatory regions of ColE1 that are involved in determination of plasmid copy number.. Proc Natl Acad Sci U S A.

[34] Churchward G, Estiva E, Bremer H (1981). Growth Rate-Dependent Control of Chromosome Replication Initiation in Escherichia Coli.. Journal of Bacteriology.

